# Alternative Splicing: Emerging Roles in Anti-Aging Strategies

**DOI:** 10.3390/biom15010131

**Published:** 2025-01-15

**Authors:** Lingyue Gao, Rong Jia

**Affiliations:** State Key Laboratory of Oral & Maxillofacial Reconstruction and Regeneration, Key Laboratory of Oral Biomedicine Ministry of Education, Hubei Key Laboratory of Stomatology, School & Hospital of Stomatology, Wuhan University, Wuhan 430072, China; gao_lingyue@whu.edu.cn

**Keywords:** alternative splicing, aging, splicing factor, anti-aging

## Abstract

Alternative splicing plays a fundamental role in gene expression and protein complexity. Aberrant splicing impairs cell homeostasis and is closely associated with aging and cellular senescence. Significant changes to alternative splicing, including dysregulated splicing events and the abnormal expression of splicing factors, have been detected during the aging process or in age-related disorders. Here, we highlight the possibility of suppressing aging and cellular senescence by controlling alternative splicing. In this review, we will summarize the latest research progress on alternative splicing in aging and cellular senescence, discuss the roles and regulatory mechanisms of alternative splicing during aging, and then excavate existing and potential approaches to anti-aging by controlling alternative splicing. Novel therapeutic breakthroughs concerning aging and senescence entail a further understanding of regulating alternative splicing mechanically and accurately.

## 1. Introduction

Aging or cellular senescence is a natural retrogressive change, characterized by degeneration in cell morphology and physiological function [1]. Aged cells feature weakened proliferation and regeneration potentials and increased cell death [2]. Aging is susceptible to a variety of chronic diseases, ranging from cardiovascular disease and neurodegenerative diseases to cancers [3].

Alternative splicing is a fundamental biological process in gene expression that allows a single gene to encode multiple proteins [4]. It occurs during the post-transcriptional modification of pre-mRNA (precursor messenger RNA) in eukaryotes. This process involves the selection of different combinations of exons (expressed regions of the gene) and introns (non-coding regions that are removed) to create various mature mRNA transcripts from a single gene. These different mRNA transcripts can then be translated into distinct protein isoforms, each with potentially unique functions, localizations, or regulatory properties [5].

**Figure 1:**
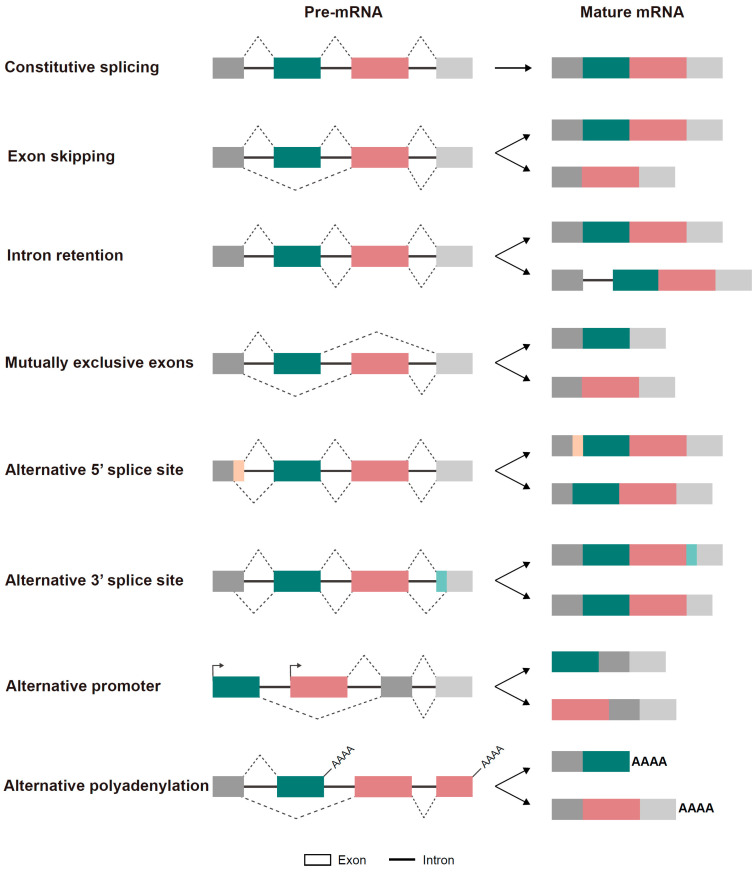
Types of alternative splicing.

Diverse types of alternative splicing events have been identified, including exon skipping (exon(s) is/are selectively excluded from the mature mRNA transcript), mutually exclusive exons (only one exon of two or several exons is included in the mature mRNA), alternative 3′ and 5′ splice site selection (different splice sites at the start or end of an exon are used, resulting in exons with variable lengths), an alternative promoter (influencing the 5′ end of mRNA through shifted transcription initiation), alternative polyadenylation (creating diverse 3′ termini), and intron retention (an intron retained in the mature mRNA) [6] (Figure 1).

RNA processing fidelity is preserved at various stages from transcription and splicing to translation in normal physiological conditions. As aging and cellular senescence occur, disruptions in RNA homeostasis can impair cellular and tissue function, potentially contributing to the development of age-related diseases. Changes in splicing factor expression [7], modifications that lead to the mislocalization of splicing factors [8], spliceosome dysfunction [9], and aberrant splicing [10], can give rise to the dysregulation of RNA homeostasis.

This raises the question: is it possible to suppress aging and cellular senescence by controlling alternative splicing? In this review, we will summarize the latest research progress on alternative splicing in aging and cellular senescence, discuss the regulatory mechanisms of alternative splicing during aging, and excavate potential approaches to anti-aging by controlling alternative splicing.

## 2. The Significant Changes in Alternative Splicing During Aging and Cellular Senescence

Aging can lead to aberrant alternative splicing among various species and tissues, characterized by dysregulated splicing events and the abnormal expression of splicing-related proteins. A meta-analysis of senescence-associated alternative splicing profiles in different types of in vitro-cultured human cells, including IMR90 (a human lung fibroblast), WI38 (a human lung fibroblast), HFF (a human foreskin fibroblast), BJ (a human foreskin fibroblast), and human fetal astrocytes, identified 406 cellular senescence-associated splicing events, among which most splicing events were exon skipping, followed by intron retention [11]. This study also demonstrated that many RNA-binding proteins were downregulated, including splicing factors serine/arginine splicing factor 1 (SRSF1), serine/arginine splicing factor 7 (SRSF7), quaking (QKI), RNA-binding Fox-1 homolog 2 (RBFOX2), polypyrimidine tract-binding protein 1 (PTBP1), heterogeneous nuclear ribonucleoprotein K (HNRNPK), heterogeneous nuclear ribonucleoprotein M (HNRNPM), and heterogeneous nuclear ribonucleoprotein U-like 1 (HNRNPUL1) [11].

The downregulation of SRSF1 in senescent cells was further confirmed in TIG-3 cells, a normal human diploid fibroblast cell line [12]. In addition, in aged human oocytes, the global translation efficiency was altered, which might be attributed to the differently expressed splicing factors such as serine/arginine splicing factor 6 (SRSF6), heterogeneous nuclear ribonucleoprotein H 1 (HNRNPH1), cleavage stimulation factor subunit 2 (CSTF2), and CUGBP Elav-like family member 1 (CELF1) [13]. In mouse oocytes, DEAD-box helicase 5 (DDX5), heterogeneous nuclear ribonucleoprotein C (HNRNPC), heterogeneous nuclear ribonucleoprotein K (HNRNPK), heterogeneous nuclear ribonucleoprotein A1 (HNRNPA1), and splicing factor proline- and glutamine-rich (SFPQ) were identified as spliceosome-associated proteins, contributing to the dysregulation of alternative splicing during the postovulatory aging process [14]. In addition, in the female mouse hippocampus, the expression of RNA-binding proteins cold-inducible RNA-binding protein (CIRBP) and RNA-binding motif protein 3 (RBM3) showed a sharp decrease in old mice [15]. Recently, more aging-related alternative splicing events have been revealed. In aged mouse testes, many non-coding RNAs show remarkable alternative splicing changes, such as *4930555F03Rik*, *1700022E09Rik*, *Gm32828*, *Gm12637*, and *1700010J16Rik* [16]. In the mouse hippocampus, there are 591 age- or sex-related alternative splicing events in 452 genes, of which the genes myelin associated glycoprotein (*Mag*), brain-enriched myelin associated protein 1 (*Bcas1*), etc., with exon skipping events have been identified as sex-independent and age-dependent AS events [17].

Aberrant alternative splicing is pertinent to various age-related disorders. Parkinson’s disease (PD) is an age-related neurodegenerative disorder causing dysfunctions in the motor system, cognition, and emotion. A transcriptome analysis of substantia nigra extracted from PD cases and healthy controls detected a novel splicing isoform of the parkinsonism-associated deglycase (*PARK7*, also called DJ-1) gene, DJ-1ΔE6, which underwent exon 6 skipping [18]. The DJ-1ΔE6 caused damage to mitochondrial function and impaired antioxidant capability, thus promoting PD development [18]. An increase in PSV2, an exon 5-skipped splicing isoform of presenilin 2 (*PSEN2*), has been detected in brain tissue from sporadic Alzheimer’s disease (AD) patients [19]. PSV2-overexpressed human neuroblastoma cells are more susceptible to hypoxia and endoplasmic reticulum stresses [19]. Therefore, the profiles of alternative splicing and the expression of splicing factors undergo significant change during aging.

## 3. Alternative Splicing of Genes Associated with Aging and Cellular Senescence

**Figure 2:**
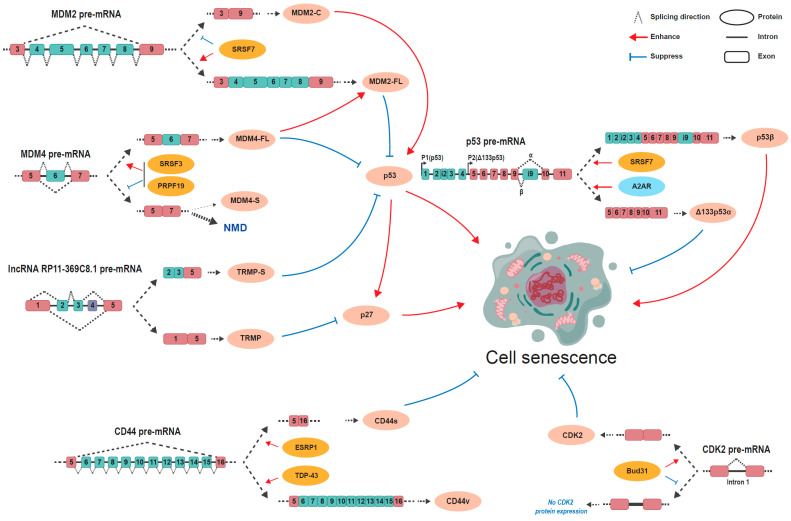
The alternative splicing of the pre-mRNAs of MDM2, MDM4, p53, CD44, CDK2, and lncRNA RP11-39C8.1 regulates cell senescence.

Even though many alternative splicing events significantly change during aging or cellular senescence, so far, only a small number of these events have been explored in detail, particularly the events or genes known to be associated with aging or cellular senescence (Table 1) (Figure 2).

### 3.1. TP53

The p53 gene holds high fidelity in organisms over time [37,38], and is located on human chromosome 17p13.1 and consists of 13 exons [39]. The human *TP53* gene produces multiple isoforms through the combination of alternative promoters (P1 and P2), alternative splicing (intron 2 and 9), and the alternative initiation of translation. p53 mediates cellular senescence via the joint effect of co-expressed p53 isoforms [20,38,39]. p53β is a splicing variant of p53 that lacks the carboxy-terminal oligomerization domain and uses the proximal 3′ splice site of exon i9 [40]. The upregulated expression of p53β has been detected in senescent cells and has been demonstrated to facilitate cellular senescence [21,22], as p53β expression promotes a cellular senescent phenotype manifested by G0/G1-phase growth arrest, SA-β-galactosidase (β-gal) positivity, senescence-associated secretory phenotype (SASP) induction, and p16INK4A induction [23]. Ionizing radiation (IR) induces senescence by suppressing the activity of SMG1, a kinase associated with nonsense-mediated mRNA decay, and allowing ribosomal protein L26 (RPL26) and the splicing factor SRSF7 to combine p53 pre-mRNA, which consequently generates the alternative splicing isoform p53β [21,23]. Δ133p53α is an N-terminally truncated anti-senescent splicing variant of p53 that uses the P2 promoter [20]. It has been observed that Δ133p53α hinders the combination of full-length p53 with p53 target genes that enhance cellular senescence in human fibroblasts [24]. The activation of the adenosine A2A receptor (A2AR) causes a decrease in full-length p53 and an increase in Δ133p53α, which helps chondrocytes fight against cellular senescence and facilitates cartilage regeneration [20].

### 3.2. MDM2/MDM4

MDM2 belongs to the RING finger protein family and is a negative regulator of the p53 pathway that ubiquitylates the p53 protein and leads to its degradation [41]. The human MDM2 gene exhibits complicated alternative splicing and has around 70 splice variants [42]. Variant MDM2-C is characterized by exon 4–8 skipping which results in a lack of the p53 binding domain in the encoded protein [25]. MDM2-C can bind to the full-length MDM2 protein and inhibit its function in p53 ubiquitination and degradation, and thus stabilizes the p53 protein. MDM2-C overexpression delays cell proliferation and induces senescence in the human diploid fibroblast. SRSF7 is the major splicing factor responsible for suppressing the skipping of exons 4–8. The knockdown of SRSF7 significantly increases MDM2-C and induces senescence in the human diploid fibroblast [25].

MDM4 also belongs to the RING finger protein family. Its structure is similar to MDM2 but without E3 ubiquitin ligase activity. It can promote MDM2-mediated degradation of the p53 protein by enhancing the E3 ubiquitin ligase activity of MDM2 [12]. *MDM4* has two splicing variants produced by the alternative splicing of exon 6, full-length MDM4-FL, and exon 6-skipped MDM4-S. MDM4-S mRNA is unstable and a target of nonsense-mediated decay (NMD) due to a premature stop codon. Senescent cells express more MDM4-S than younger cells. SRSF3 can enhance *MDM4* exon 6 inclusion and the expression of the full-length MDM4 variant [26]. Recently, a study found that another splicing factor PRPF19 was downregulated in senescent normal human diploid fibroblasts and is required for the inclusion of *MDM4* exon 6. The knockdown of PRPF19 decreases MDM4-FL, increases the p53 protein level, and induces cellular senescence [27].

### 3.3. lncRNA RP11-369C8.1

The lncRNA *RP11-369C8.1* gene is a long non-coding RNA and has at least six alternative splicing variants [28]. TRMP (a TP53-regulated modulator of p27) and TRMP-S have been identified as two critical splicing variants of lncRNA *RP11-369C8.1* that promote cell proliferation and inhibit cellular senescence in a p27-dependent manner [28,29]. TRMP skips exons 2–4 and functions as a TP53-induced suppressor of the internal ribosomal entry site (IRES)-dependent p27 translation by competing for binding to p27 mRNA with polypyrimidine tract-binding protein 1 (PTBP1) [29]. TRMP-S skips exon 1 and 4 and performs a different mechanism to restrain p27 [28]. At the transcriptional level, TRMP-S impairs the level of p27 mRNA by stabilizing its epigenetic inhibitor UHRF1 (an E3 ubiquitin ligase) [28]. At the translational level, a combination of TRMP-S and FUBP3 (far upstream element-binding protein 3) keeps the RPL26 ribosomal protein from binding to p53, causing a decreased p53 translation and restrained p27 expression [28].

### 3.4. CD44

CD44 is a transmembrane proteoglycan belonging to the cartilage link protein family [43,44], whose primary ligand is hyaluronan (HA) [45]. *CD44* consists of 19 exons and 20 exons, respectively, in humans and in mice. The alternative splicing of *CD44* generates the CD44s (standard) isoform and the CD44v (variant) isoform. The CD44s comprises exons 1–5 and 16–20, which are standard exons, while the CD44v contains extra variable exons 6–15 on the basis of CD44s [46,47]. The abundance of CD44v maintains the stemness of breast cancer stem cells (BCSCs) [48]. TDP-43 facilitates the shift from CD44s to CD44v through the inclusion of variable exons, especially exon v8–v10, and thus stabilizes the BCSCs’ stemness [30]. However, another study has reported that CD44s maintains the cellular plasticity of BCSCs concerning differentiation, while CD44v functions inversely [49]. Shifting from CD44v to CD44s regulated by the splicing factor epithelial splicing regulatory protein 1 (ESRP1) induces the BCSCs’ features [49]. An increase in CD44s and a decrease in CD44v regulated by ESRP1 perform an anti-senescence effect on human amniotic epithelial stem cells (hAECs) by activating the AKT/mTOR signaling pathway [31]. The complicated mechanism of *CD44* alternative splicing in different cell stages and cell types might underlie the deceptive discrepancy listed above.

### 3.5. CDK2

Cyclin-dependent kinase 2 (CDK2) is a member of the CDK family, a cell proliferation regulator which keeps the cell cycle progression from stagnating in the G1 phase [50,51]. *CDK2* has two known splicing variants, CDK2-β (a full-length isoform) and CDK2-α (an exon 6-skipped isoform), regulating G1/S transition and the early S phase during cell proliferation [51]. Aberrant splicing events such as the intron retention of *CDK2* pre-mRNA result in a decrease in CDK2 protein expression and consequently promote cellular senescence [32,33]. PHD finger protein 5A(PHF5A) K25 decrotonylation induced by SIRT7 and the depletion of Bud31 have been demonstrated to facilitate the retention of intron 1 [32,33].

### 3.6. SIRT1

SIRT1 (sirtuin 1) is a member of the sirtuin protein family, which is an NAD^+^-dependent deacetylase that couples target substrate deacetylation with cellular metabolic status [52,53]. The human *SIRT1* gene is located on chromosome 10 and bears 11 exons in the genomic region, generating at least three splicing variants (SIRT1-v1, SIRT1-v2, and SIRT1-v3) [34]. SIRT1-v1 is the longest of these, containing nine exons, while SIRT1-v2 contains eight exons with exon-1 and exon-3 skipped and an extra exon (exon-1′) included, and SIRT1-v3 contains seven exons with exon-1, -2, and -3 skipped and an extra exon (exon-4′) included [34]. As a result of exon skipping, the two shorter isoforms SIRT1-v2 and SIRT1-v3 lack a nuclear localization signal and carry a shortened N-terminal region, which not only affects the protein’s intracellular localization but also undermines the protein–protein interactions and biological processes especially deacetylation mediated by SIRT1 [34]. A dynamic expression of SIRT1 variants in human hearts during aging has been observed. SIRT1-v1 expression peaks in fetal hearts, and decreases in 24-, 72-, and 102-year-old hearts, while SIRT1-v2 and SIRT1-v3 expression are low in fetal hearts, but show an increasing tendency in 24-and 72-year-old hearts, and then decline again in 102-year-old hearts [34]. Unlike humans, mice only have two splicing variants, the authentic SIRT1-v1 and the shorter variant SIRT1-v2 that lacks exon 2 [34]. Stress in adolescence causes a tendency toward the shorter isoform of SIRT1 both at the mRNA expression level and at the protein level in the mouse cortex, which might impair cognition based on a senescence-related mechanism [35]. SIRT1 has been identified as a promising anti-senescence regulator and improves longevity in various organisms [35,54]. For example, in prolonged culture of primary endothelial cells isolated from porcine aorta, SIRT1 promoted cell proliferation and inhibited senescence by inactivating the LKB1-activated AMPK pathway [36]. It is possible that shorter isoforms of SIRT1 may contribute to cellular senescence and age-related disorders.

## 4. Controlling Aging or Cellular Senescence by Key Splicing Factors

**Figure 3:**
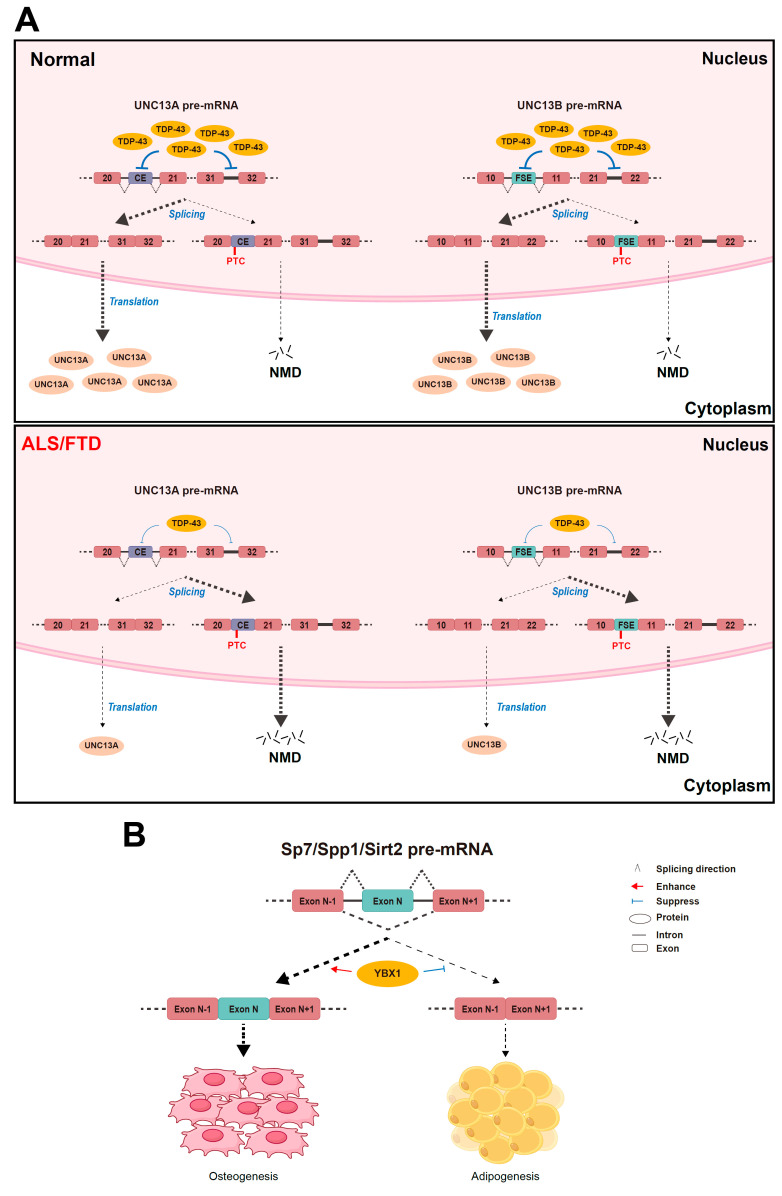
Controlling aging or cellular senescence by key splicing factors. (**A**) Novel exon inclusion or intron retention impaired the expression of UNC13A or UNC13B proteins which are underexpressed in amyotrophic lateral sclerosis (ALS) and frontotemporal dementia (FTD). CE represents cryptic exons. FSE represents the frameshift exon. NMD represents nonsense-mediated decay. (**B**) YBX1 promotes osteogenic differentiation and represses aging-associated adipogenic differentiation in bone marrow stromal cells by suppressing the exon skipping of the *Sp7*, *Spp1*, and *Sirt2* genes.

Alternative splicing is mainly regulated by splicing factors which may play key roles during aging and cellular senescence (Figure 3).

### 4.1. TDP-43

Transactivation response element DNA-binding protein 43 (TDP-43) is an RNA-binding protein that is mainly located in the nucleus and plays an essential regulating role in alternative splicing, including acting as a splicing repressor of cryptic exon inclusion and retaining intron integrity [55,56,57]. In mouse embryonic stem cells, the knockout of TDP-43 helps cryptic exons to be spliced into mRNAs, resulting in frame shifts and premature termination, and even impairing RNA stability [55].

*UNC13A* is one of the genes that undergo significant mis-splicing upon the depletion of TDP-43. In neuronal cell lines and motor neurons derived from human-induced pluripotent stem (iPS) cells, the loss of TDP-43 in the nucleus leads to the insertion of a 128 bp or a 178 bp cryptic exon (CE) between the standard exons 20 and 21 as well as increased intron retention between exons 31 and 32 in *UNC13A* mRNA, and introduces premature stop codons causing nonsense-mediated decay and downgraded UNC13A protein levels [56,57]. A lack of the UNC13A protein impairs synapse function and underlies the pathological mechanism of neurodegenerative diseases including amyotrophic lateral sclerosis (ALS) and frontotemporal dementia (FTD) [56]. Another member of the UNC13 synaptic protein family, UNC13B, undergoes the inclusion of a frameshift exon (FSE) between exons 10 and 11 and an increase in intron retention between exon 21 and 22 upon TDP-43 knockout in an analogical manner in iPS cell-derived cortical-like neuron cells [57]. Like *UNC13A* and *UNC13B*, three other synapse-related genes *KALRN*, *RAPGEF6*, and *SYT7* are also susceptible to abnormal splicing in the context of TDP-43 depletion [56]. Generally, the depletion of TDP-43 contributes to neurodegenerative diseases like ALS and FTD (Figure 3).

TDP-43 regulates the alternative splicing of *TARDBP* mRNA that encodes the TDP-43 gene itself and contributes to the auto-regulation of the amount of nuclear TDP-43 [58]. Damage to the auto-regulating mechanism of TDP-43 has been speculated to underlie ALS/FTD pathogenesis [59]. Hypothetically, aging-related DNA methylation impairs TDP-43 autoregulation [59] and the underlying mechanism remains unknown and is worth researching.

### 4.2. YBX1

The Y-box binding protein 1 (YBX1) is a member of the cold shock domain (CSD) protein superfamily [60], which is composed of an N-terminal Ala/Prorich domain (A/P), a central CSD, and a C-terminal domain (CTD) consisting of basic and acidic clusters [61,62]. Previous studies have shown that YBX1 takes part in diverse cellular functions, including DNA repair, regulation at the transcription level, pre-mRNA splicing, mRNA packaging, and the regulation of mRNA translation and stability [63,64].

YBX1 plays a predominant role in the differentiation and senescence of bone marrow stromal cells (BMSCs). A combination of RNA sequencing and anti-YBX1 cross-linking immunoprecipitation–high-throughput sequencing analysis identified BMSC osteogenesis-related genes *Fn1*, *Sp7*, and *Spp1*, and BMSC senescence-related genes *Sirt2* and *Nrp2* as direct YBX1 mRNA-binding targets in a BMSC cell line [65]. In mouse BMSCs, the knockout of YBX1 leads to mis-splicing, including the exon skipping of those genes [65]. The full-length isoforms of *Sp7*, *Spp1*, and *Sirt2* could reverse the inhibited osteogenesis differentiation and the growing adipogenic differentiation, and rescue the senescence of BMSCs caused by YBX1 depletion [65]. Hence, YBX1 could enhance osteogenesis differentiation and suppress the senescence of BMSCs by regulating the alternative splicing of relative genes [65].

YBX1 can stimulate the inclusion of *CD44* exon v5 depending on its binding to the core high-affinity motif CAUC [61]. The dimerization of the CSD contributes to its RNA-binding affinity and the regulation of alternative splicing to a great extent [62]. The disruption of the dimeric interface has been reported to impair the *CD44* exon v5 splicing in a human breast cancer cell line stably transfected with a *CD44* minigene system [62]. Accordingly, YBX1 participates in the alternative splicing of the key age-related gene *CD44*; its specific role in regulating aging and cellular senescence through *CD44* variants, however, remains unclear.

### 4.3. Other Splicing Factors Controlling Aging or Cellular Senescence

Some other splicing factors also show the potential roles of anti-aging or senescence. For example, SRSF3 knockdown caused cellular senescence through increasing p53β expression in normal human fibroblast [66]. Serine/arginine-splicing factor 11 (SRSF11) expression significantly decreases in the prefrontal cortex tissues of 24-month-old mice compared with 2-month-old mice. Mice with SRSF11 knockdown showed aging-associated cognitive decline due to the activation of the c-Jun N-terminal kinase (JNK) signaling pathway [67]. The overexpression of the senescence evasion factor (SNEV), a splicing factor involved in the assembly of the spliceosome [68], significantly extends the life span of human endothelial cells by improving DNA repair [69]. Intriguingly, some splicing factors have dual roles in aging or senescence. For example, SRSF1 knockout induces cellular senescence in mouse muscle stem cells [70]. However, another study showed that SRSF1 overexpression actually induced senescence in primary human fibroblast [71]. These studies suggest that splicing factors play essential roles during aging or senescence. However, their roles may vary in different types of cells or tissues.

### 4.4. Selection of Key Splicing Factors

Splicing factors control the alternative splicing of a number of target genes with cell- or tissue-type specificity. It is essential to individually evaluate their roles during aging or senescence in different types of cells or tissues. In addition, some splicing factors have been demonstrated to act as oncoproteins [72]. Although the deficiency of some splicing factors often causes senescence, overexpressing these splicing factors may induce cell transformation. Therefore, a potential anti-aging method via modifying the expression of splicing factors and globally changing the cellular splicing profiles should avoid oncogenic splicing factors.

## 5. Anti-Aging by Controlling Alternative Splicing

**Figure 4:**
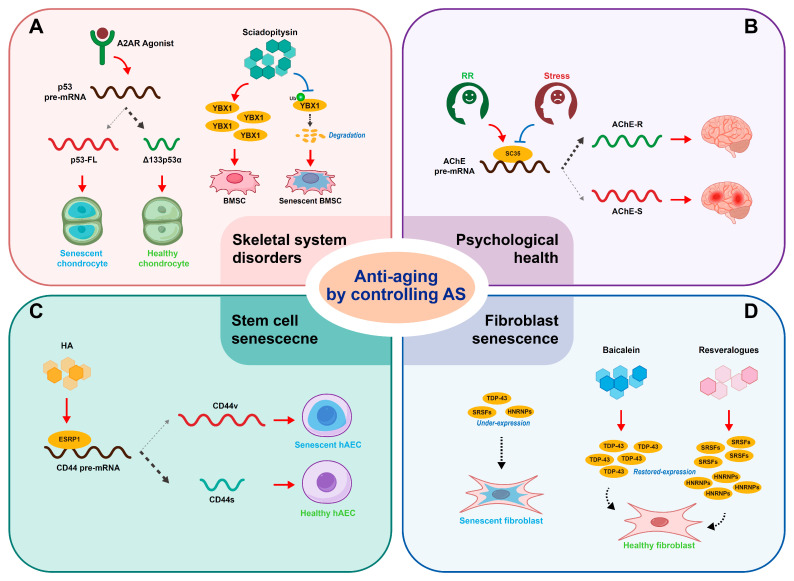
The methods of anti-aging by controlling alternative splicing in skeletal system disorders (**A**), psychological health (**B**), stem cells (**C**), and fibroblasts (**D**).

Given the emerging regulatory roles of alternative splicing during aging, a number of methods of anti-aging by controlling alternative splicing have been introduced recently (Figure 4).

### 5.1. Anti-Aging of Skeletal System

In primary or immortalized human chondrocytes, A2AR activation causes a decrease in full-length p53 and an increase in Δ133p53α, which helps chondrocytes fight against cellular senescence and facilitates cartilage regeneration [20]. The finding has indicated that the A2AR agonist is a prospective therapeutic method for chondrocyte senescence and closely relevant osteoarthritis.

A natural small molecular compound, Sciadopitysin, appears to control the senescence of BMSCs by targeting YBX1-related alternative splicing. A recent study suggested that Sciadopitysin could occupy the crucial pocket-like site for YBX1 combining with ubiquitin ligase FBXO33 and decrease the FBXO33 level to prevent YBX1 from binding to FBXO33 [65]. FBXO33 has been indicated to associate with YBX1 and subjects YBX1 to ubiquitination-mediated degradation in HEK293T cells [73]. Therefore, Sciadopitysin was supposed to rescue YBX1 expression. In addition, Sciadopitysin could inhibit the exon skipping of *Sirt2*, *Fn1*, and *Spp1* to some extent in BMSCs isolated from 24-month-old mice [65]. Overall, Sciadopitysin provided the possibility of anti-senescence treatment for age-related osteoporosis by targeting mis-splicing in aged BMSCs [65].

### 5.2. Anti-Aging of Psychological Health

Stress has been suggested to accelerate biological aging [74], while the benefits of psychological intervention concerning aging require further investigation. Stress-induced upregulation in miRNAs miR-134 and miR-183 has been demonstrated to impair cholinergic neurotransmission by downregulating splicing factor SC35 [75] since SC35 could help switch the alternative splicing of acetylcholinesterase (AChE) from the isoform associated with the synapse (AChE-S) to a soluble one (AChE-R) [76]. The practice of a relaxation response (RR) appeared to reverse the stress-induced effect, given the significant decrease in miR-134 and miR-183 in the sera of both patients with ischemic heart disease and healthy controls treated with RR [77].

### 5.3. Anti-Aging of Stem Cells

It has been reported that a 300 kDa hyaluronic acid (HA) treatment inhibits the cellular senescence of hAECs [31]. Mechanically, as a splicing factor, ESRP1 increases under HA treatment and switches the CD44v isoform to the CD44s isoform [31]. As a result, an increase in CD44s and a decrease in CD44v inhibits the senescence of hAECs [31].

### 5.4. Anti-Aging of Fibroblasts

Senescent human primary fibroblasts feature reduced mRNA expression of splicing factors [78]. Recovery of splicing factor expression was likely to reverse the cellular senescence [78]. A study demonstrated that small molecule resveralogues could reverse the age-related underexpression of splicing factors by elevating both splicing activators (SRSFs) and inhibitors (HNRNPs), and could improve the senescence phenotypes in senescent fibroblasts [78]. Likewise, trametinib and SH-6 have been demonstrated to serve a similar function of elevating the expression of multiple splicing factors including SRSFs and HNRNPs through the inhibition of ERK or AKT signaling in senescent human primary fibroblasts [79]. Additionally, the direct knockout of the ERK and AKT downstream effector genes ETV6 and FOXO1 could also make a contribution to restoring the expression of splicing factors and mitigating cell senescence [79].

In the context of progeroid syndromes, such as Hutchinson Gilford Progeroid syndrome (HGPS), Werner syndrome (WS), and Cockayne syndrome (CS), fibroblast cells have been characterized by accelerated cellular senescence and misregulated splicing factor expression, including the downregulation of most splicing factors, similar to normal senescent fibroblast cells [80]. Trametinib could rehabilitate the responsiveness of splicing factor expression and mitigate the senescent burden in human dermal fibroblasts (HDFs) under an HGPS and CS context, suggesting that trametinib and other senomorphic drugs might serve as a therapeutic possibility for progeroid diseases [80]. Moreover, in progeria cells, TDP-43 suffers dysfunction that fails to promote CFTR exon 9 skipping [81]. Baicalein can rescue the activity of TDP-43 as well as restore the retention of nuclear TDP-43 [81]. Therefore, baicalein has been identified as a potential therapeutic agent for age-associated diseases.

These anti-aging therapies that control alternative splicing are summarized in Table 2.

### 5.5. Suppressing the Risk of Oncogenesis

Many aging-related genes are tumor suppressors or oncogenes. The suppression of a tumor suppressor or the upregulation of an oncogene may increase the risk of cancer. For example, p53-knockout mice spontaneously developed a variety of neoplasms [82] and the inactivation of p53’s tumor-suppressive function led to human cancers [83]. Therefore, more research may be required to explore a precisely regulated anti-aging strategy by balancing the ratio of the anti-senescence isoforms to the pro-senescence isoforms of an aging-related gene to lower the risk of cancer.

### 5.6. Restoring the Alternative Splicing Profile of Young Cells

The alternative splicing profiles in aged cells is significantly different from young cells [84]. An anti-aging therapeutic effect may be achieved by switching the alternative splicing profiles of aged cells to those of young cells. The overexpression of splicing factors downregulated in senescent cells may globally restore the alternative splicing profiles of young cells. In addition, most studies have focused on a single gene or event. However, simultaneously modifying the alternative splicing events of multiple aging-related genes may achieve relatively better anti-aging effects.

## 6. Conclusions and Remarks

In conclusion, the alternative splicing of aging-associated genes produces anti-aging or pro-aging isoforms. Enhancing the expression of an anti-aging isoform and suppressing the expression of a pro-aging isoform may pave the way for the treatment of aging and aging-related diseases.

Alternative splicing is an essential molecular regulatory mechanism of gene expression. In general, alternative splicing profiles show relative homeostasis in cells. The disturbance of cellular alternative splicing profiles may induce cell apoptosis and senescence. Therefore, it is important to specifically control the key alternative splicing events related to aging without interfering with the whole alternative splicing profile of cells.

Aging-associated genes are often “gatekeepers” of the genome’s stability and act against cancers. The p53 pathway is an essential break to prevent tumors. p53-knockout animals suffer from a variety of spontaneous cancers. Therefore, any anti-aging method that interferes with the p53 pathway, such as by modifying the alternative splicing of p53, should carefully avoid destroying the tumor-suppressive function of p53. The inactivation of p53 may not be feasible; however, the reversible adjustment of p53 isoforms by relatively increasing an anti-aging isoform such as Δ133p53α or decreasing a pro-aging isoform p53β may provide a new avenue for anti-aging treatment. Other genes associated with both aging and tumors or associated with p53 may also be suitable for an alternative splicing adjustment instead of full inactivation or activation.

The effect of an aging-related gene on aging and senescence is based on the integrated expression of all isoforms. Different isoforms of one gene may play commensal, synergic, or exclusive roles in the aging process. SIRT1 has been identified as a promising anti-aging regulator, while three isoforms of *SIRT1* show a dynamic expression in human hearts during aging [34]. A trend towards its shorter isoforms may accelerate senescence, whose fundamental mechanism remains unknown [35]. It is of great importance to explore the specific function of each isoform and then regulate the splicing events more accurately.

Given the complicated mechanism of alternative splicing and aging, a splicing isoform may have a seemingly inconsistent impact on aging, that is, it may act as both an aging promoter and inhibitor. The shift from CD44s to CD44v induces BCSC features including longevity [48] and vice versa [49]. One possible reason may be the diversity of the CD44v isoform as it contains one to several extra variable exons from exon 6 to exon 15, performing various functions. And the discrepancy between normal cells and cancer cells may partially explain the contradiction. Whether a splicing isoform plays a pro-aging role or an anti-aging role depends not only on the isoform itself but also on the cell type and cell stage to a great extent.

Alternative splicing offers a novel and promising perspective towards anti-aging strategies. Increasing treatments for the senescence of different kinds of cells, like stem cells and fibroblasts, and age-related disorders concerning different systems, like the skeletal system and the neurological system, are emerging. It is not impossible to reverse the senescence of specific cells through the precise regulation of alternative splicing.

## Figures and Tables

**Table 1:** Alternative splicing events associated with aging or cellular senescence.

Gene Name	Splice Variants	Exon Location	Splicing Events	Roles in Aging	Regulatory Mechanism	Refs.
*TP53*	p53β	Exon i9	Alternative 3′ splice site	Promoting cellular senescence	RPL26–SRSF7 promotes the p53β isoform	[20,21,22,23,24]
	Δ133p53α	P2 promoter	Alternative promoter	Inhibiting cellular senescence	A2AR promotes the Δ133p53α isoform	
*MDM2*	MDM2-C	Exons 4–8	Exon skipping	Promoting senescence	SRSF7 inhibits the skipping of exons 4–8	[25]
*MDM4*	MDM4-FL and MDM4-S	Exon 6	Exon skipping	MDM4-FL suppresses senescence	SRSF3 and PRPF19 inhibit the skipping of exon 6	[26,27]
*lncRNA RP11-369C8.1*	TRMP	Exons 2–4	Exon skipping	Promoting cell proliferation and inhibiting cellular senescence	N/A	[28,29]
	TRMP-S	Exons 1 and 4	Exon skipping			
*CD44*	CD44s	exons 1–5 and 16–20	Standard exons	Inhibiting hAECs senescence	ESRP1 upregulates the CD44s and downregulates the CD44v TDP-43 promotes the inclusion of variable exons	[30,31]
	CD44v	exons 6–15	Variable exons	Promoting hAECs senescence		
*CDK2*	N/A	Intron 1	Intron retention	Promoting senescence	PHF5A K25 decrotonylation and the depletion of Bud31 promote the retention of intron 1	[32,33]
*SIRT1*	SIRT1-v1	N/A	N/A	Inhibiting senescence and improving longevity	N/A	[34,35,36]
	SIRT1-v2	Exons 1 and 3	Exon skipping	Promoting senescence		
		Exon 1′	Exon inclusion			
	SIRT1-v3	Exons 1, 2, and 3	Exon skipping			
		Exon 4′	Exon inclusion			

N/A: Not applicable

**Table 2:** Anti-aging therapies that control alternative splicing.

Aging-Related Disorders		Splicing Events or Splicing Regulators	Anti-Aging Methods	Refs.
Skeletal system	Senescence of chondrocytes and osteoarthritis	Decrease in full-length p53 Increase in Δ133p53α	A2AR agonist	[20]
	Senescence of BMSCs and age-related osteoporosis	Exon skipping of BMSC osteogenesis-related and senescence-related genes	Sciadopitysin	[65]
Psychological health		Increase in SC35	Relaxation response	[81]
Stem cells	Senescence of hAECs	Increase in CD44s and decrease in the CD44v regulated by ESRP1	Hyaluronic acid	[31]
Fibroblast		Increase in SRSFs and HNRNPs	Resveralogues, trametinib, and SH-6	[78,79]
		TDP-43-mediated exon skipping	Baicalein	[75,77]

## Data Availability

All relevant data are within the paper.
